# Offshore telementored ultrasound: a quality assessment study

**DOI:** 10.1186/s13089-020-00180-9

**Published:** 2020-07-02

**Authors:** Victoria Vatsvåg, Kjetil Todnem, Torvind Næsheim, John Cathcart, Daniel Kerr, Nils Petter Oveland

**Affiliations:** 1 School Health Sciences, Ulster University, Co., Antrim, UK; 2 Faculty of Health Sciences, Department of Quality and Health Technology, University of Stavanger, Stavanger, Norway; 3 Department of Health and Working Environment, Global Business Support, Equinor ASA, Stavanger, Norway; 4 Cardiovascular Research Group, Department of Clinical Medicine, Faculty of Health Sciences, UiT, The Arctic University of Norway, Tromsø, Norway; 5 Department of Anaesthesiology, University Hospital North Norway, Tromsø, Norway; 6 Department of Anaesthesiology and Intensive Care, Stavanger University Hospital, Stavanger, Norway

**Keywords:** Ultrasound, Telementored ultrasound, PoCUS, Offshore, Remote

## Abstract

**Background:**

Telementored ultrasound (US) connects experts to novices through various types of communication and network technologies with the overall aim to bridge the medical imaging gap between patients’ diagnostic needs and on-site user experience. The recurrent theme in previous research on remote telementored US is the limited access to US machines and experienced users. This study was conducted to determine whether telementored US was feasible in a remote offshore setting. The aim was to assess if an onshore US expert can guide an offshore nurse through focused US scanning protocols by connecting an US machine to existing videoconference units at the offshore hospitals and to evaluate the diagnostic quality of the images and cineloops procured.

**Results:**

The diagnostic quality of cineloops was scored on a five-point scale. The percentage of cineloops suitable for interpretation (score 3 ≥) for the FATE and e-FAST protocols was 96.4 and 79.1. Lung sliding and seashore sign could be identified in all volunteers. The scan time for the FAST protocol (*n* = four scanning positions), FATE protocol (*n* = six scanning positions) and both lungs (*n* = two scanning positions) was 1 min 20 s, 4 min 15 s and 32 s, respectively.

**Conclusion:**

A novice US user can be guided by a remote expert through focused US protocols within an acceptable time frame and with good diagnostic quality using existing communication and network systems found onboard offshore oil rigs.

## Background

Historically, practising medicine has always been a combination of skills for diagnosis and commencing treatment based on patients’ signs and symptoms [1, 2]. As one would expect, doing so in an austere environment is even more challenging where the clinical scenarios and patterns of illness and injury vary widely, and the access to medical equipment and qualified healthcare personnel are limited or even lacking [3]. Norway is an important supplier of oil and gas to the global market and employs approximately 21,000 people running between 80 and 90 oil installations [4]. These platforms and ships are spread along the Norwegian continental shelf and have limited medical and logistic support. Operating in such remote locations and challenging climatic environments requires a well-functioning health service [5]. Today, this remote medical practice is run by offshore nurses in hospital units onboard the installations and search-and-rescue (SAR) personnel working as part of medical evacuation (medevac) teams onboard helicopters [6]. The nurses, often working alone, are trained to carry out focused clinical examinations, perform certain medical tests, including recording vitals and electrocardiograms (ECGs), and provide simple blood and urine tests (e.g., haemoglobin, CRP, glucose). The nurse also has the opportunity to consult with a physician onshore, either by phone or videoconference [7]. However, this remote offshore healthcare service is in many cases insufficient for determining a final diagnosis [8, 9]. Whereas patients hospitalized onshore are referred for further medical imaging, such as X-ray, computerized tomography (CT), magnetic resonance imaging (MRI) or ultrasound (US), to determine their diagnosis [1, 10], this equipment has not, to date, been available for offshore workers. The result is an extended use of medevacs with SAR helicopters to bring the patients off the platforms and admit them to onshore hospitals. This process is a costly affair and not without risks, especially in challenging weather conditions [6]. It can take several hours from alerting the SAR team until the patient reaches definitive care, or in the worst-case scenario, no evacuation is possible due to restricted flying conditions [7]. Therefore, there is a need for extended medical practice with provision of more advanced diagnostic and management advice via telecommunication (i.e., telemedicine [11, 12]). A solution could be to connect US machines to the already installed videoconference units (medical units) found onboard most oil installations [13]. The development of lightweight, battery-powered, and easily transportable devices has made US in the field (i.e., outside hospitals) possible in contrast to bulkier and power-demanding X-ray machines and CT and MRI scanners [14]. Continued improvement in both US and telecommunication technology may open opportunities for improved clinical decision-making [12, 15]. US imaging can be obtained instantly and correlated to the patient’s presenting signs and symptoms and repeated if the condition changes [16]. Combining the offshore nurses’ physical examination with a focused goal-directed US scan [i.e., point-of-care ultrasound (PoCUS)] can confirm and refute life-threatening diagnoses, thereby assisting the onshore physicians in the initial evaluation and management of critically ill and injured patients [16, 17]. However, the acquisition and interpretation of PoCUS examinations are highly user-dependent [17, 18]. The operator experience amongst offshore nurses and SAR personnel thus appears to be a limitation to widespread use. We believe that this limitation can be compensated through real-time assistance using videoconferencing to link novice operators to geographically separate US experts to enable remote guidance of PoCUS examinations, an activity recognized as telementored US, tele-US or remote telementored US (RTMUS) [17, 19]. This utility of US has been evident in various extreme expeditions, such as to Mount Everest [20]. In the last decade, experience with remote applications for PoCUS has continued to increase, and research has been carried out in a variety of locations, such as rural areas in third world countries and even in space (International Space Station) [21–24]. However, telementored US between offshore nurses and an onshore on-call physician service has never been tested and evaluated. Thus, our study describes the initial experiences from a telementored US trial sending real-time video and US images from an offshore installation.

## Objectives

The objectives of this research are as follows:

- Test if the telecommunication technologies and networks available at an offshore oil installation support real-time streaming of US cineloops and images.
- Test if an onshore physician is able to instruct and guide an offshore novice user in performing pre-defined PoCUS protocols of the lungs, heart and abdomen.
- Analyse the US cineloops and images procured by novice users to determine if the quality is sufficient to extract useful clinical information.

## Methods

### Study design and setting

**Fig. 1:**
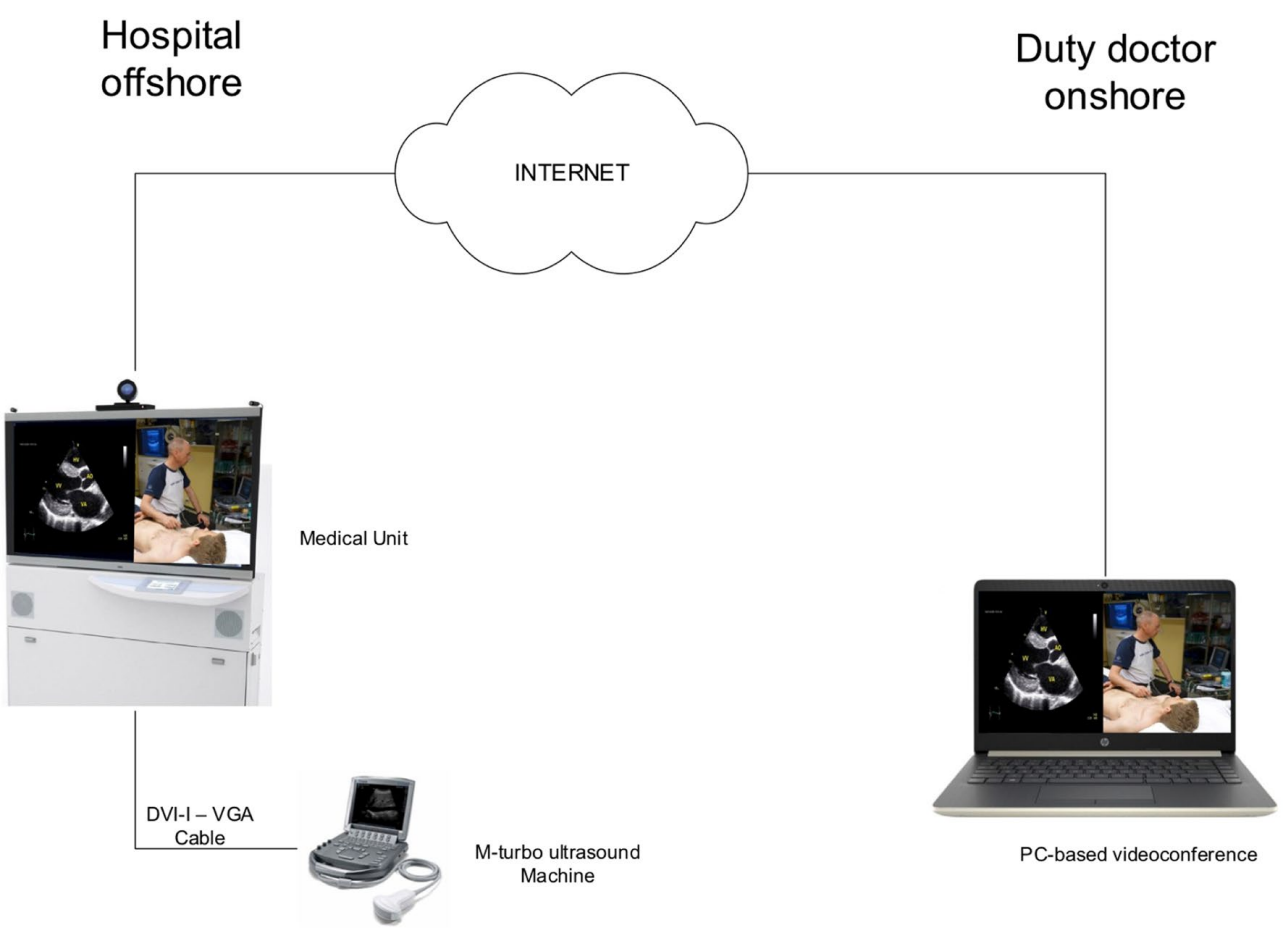
Technical setup

This study was a quality assessment study conducted in 2012 at the Statfjord C rig at the Statfjord oil field covering 580 km^2^ in the United Kingdom—Norwegian boundary of the North Sea. Statfjord C has a capacity of 345 persons on board served by one single hospital unit. The hospital is manned by one nurse, working 14-day rotations, in collaboration with onshore physicians working on-call 24-7 to assist in diagnosis and treatment decisions [13]. In most cases, the communication is via telephone (i.e., audio communication), but there is the option of using a preinstalled videoconference unit to enable video and audio from the hospital bay (i.e., the patient’s bed area) to be transmitted to remotely located computers (with preinstalled communication software) [12]. The US machine was directly connected to the medical unit and transmitted the US image and roof camera video to an onshore computer via an internet connection. The technical setup is illustrated in Fig. 1 and described in detail under “Equipment”.

### Ethical considerations

This study was exempt from formal ethical approval according to mail correspondence with the regional committees for medical and health research ethics (REK) in Norway. The study protocol was reported to the Norwegian Centre for Research Data (NSD), and considerable efforts were made to protect the interests of the participants. Participation was voluntary; we analysed the data anonymously, informed the participants about the study in writing, and notified them of their right to withdraw consent at any time. A written agreement including the invitation and participation without compensation was signed by all parties. In the case that pathology was found during the US scan, the participant was referred to his/her primary care physician. Furthermore, we also encouraged the participants to contact the researchers if they had any concerns or questions.

### Equipment

**Fig. 2:**
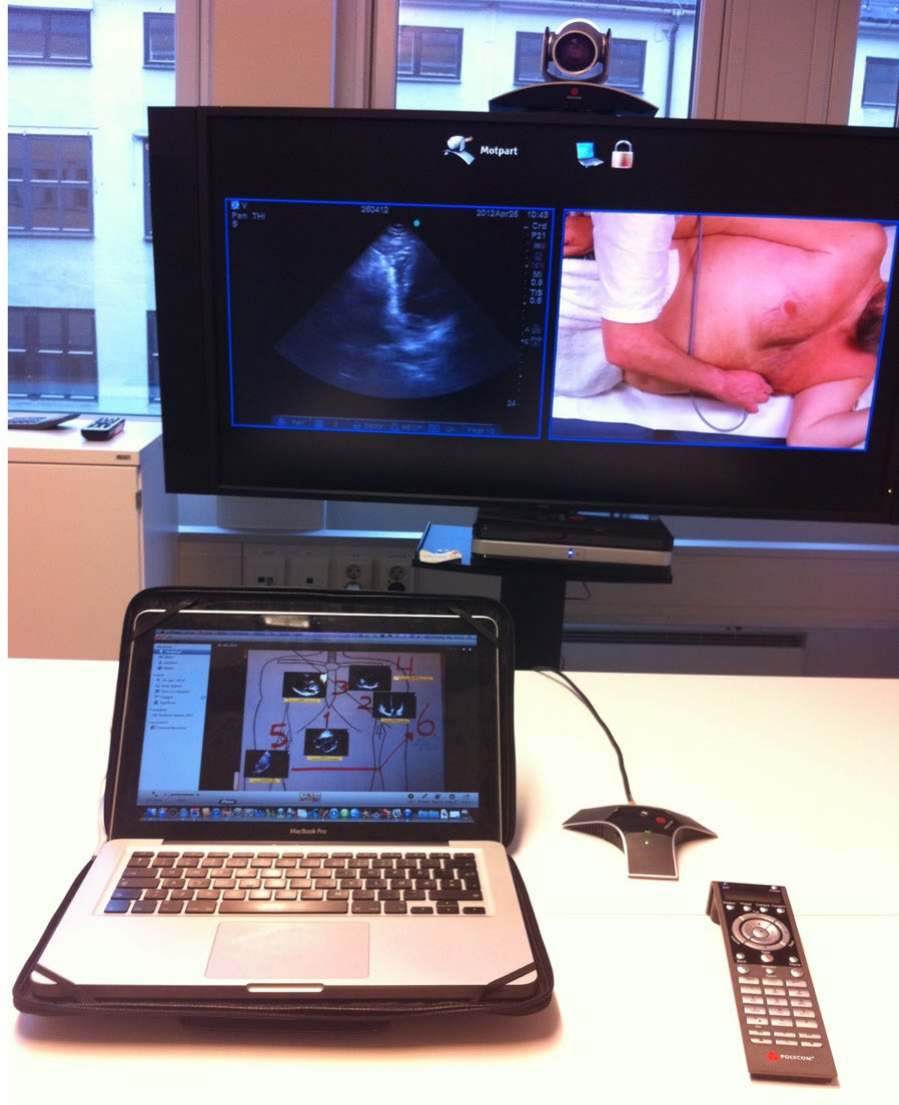
Onshore computer showing the split screen with both the ultrasound image and roof camera video. Photo: Nils Petter Oveland

An M-Turbo US machine (FUJIFILM SonoSite Inc, Bothell, WA, USA) with three bandwidth phased-array (P21x probe 5-1 MHz), curved-array (C60xi probe 5–2 MHz) and linear-array (L25x probe 13-6 MHz) transducers (FUJIFILM SonoSite Inc, Bothell, WA, USA) was connected to the videoconference unit (Cisco TelePresence C90, Cisco Systems Inc., San Jose, CA, USA) via a video graphics array (VGA) cable. The M-Turbo machine does not have a VGA outlet and was solved using a small docking station with a digital visual interface integrated (DVI-I) to VGA converter. The videoconference unit is called Medical unit and will be referred as such in the remaining text and figure legends. The VGA cable relays analogue component video signals and data from the US machine to the Medical unit, which together with the main and roof cameras (Cisco P60, Cisco Systems Inc., San Jose, CA, USA), allow real-time communication of audio–video signals between the oil installation to a Medical unit or remote computer with Cisco Jabber communication software (Cisco Systems Inc., San Jose, CA, USA) in the duty doctor’s office (Fig. 1). As a default, a 50%–50% split screen solution showing both the US image and video of the patient on the hospital bed was used (Fig. 2). The sharing of the US stream and the bidirectional sharing of audio and video were transferred live through an encrypted video stream. As shown in Fig. 1, the duo-video was transmitted to the duty doctor’s Medical unit (Fig. 2) through the corporate network at Statfjord C. All communication equipments, including servers and routers, are readily available and can be bought off the shelf. Todnem et al. [12] have described the use of medical units as an integrated part of the health service on offshore oil installations on the Norwegian continental shelf.

### Participants

The participants (*n* = 37) in the study were volunteer offshore workers that, based on availability, consented to serve as US scanning models. No volunteers were rejected from participating and the final number of 37 participants was based on the maximum capacity of a 2-day study period offshore at Statfjord C. All US scanning were performed by the offshore nurse on call (*n* = 1) and remotely guided by an onshore physician (*n* = 1) using videoconference communication as described. The nurse (i.e., novice user) had no previous US scanning experience, and the onshore physician (i.e., expert user) had more than 10-year clinical experience in PoCUS.

### Data acquisition

The nurse received an initial ½ h practical demo (i.e., knobology) of the M-Turbo US machine, including how to insert patient data and save US images and cineloops. This was done offshore and the demo was given by the research supervisor. The remotely located onshore physician then guided the nurse through two defined scanning protocols: Focused Assessed Transthoracic Echo (FATE) of the heart and pleural space [25] and extended Focused Assessment with Sonography for Trauma (e-FAST) of the abdominal, pleural and pericardial space and bilateral lung scans [26]. The FATE, FAST, e-FAST and lung protocols are extensively described in the literature [27–29]. The nurse was guided through the examination as described in the FATE protocol and obtained a 6-s cineloop from each position. For each volunteer (*n* = 37), cineloops using the phased-array cardiac probe were recorded at each of the following positions: (1) subcostal 4-chamber view, (2) apical 4-chamber view, (3) parasternal long-axis view, (4) parasternal short-axis view, (5) pleura right side and (6) pleura left side.

**Fig. 3:**
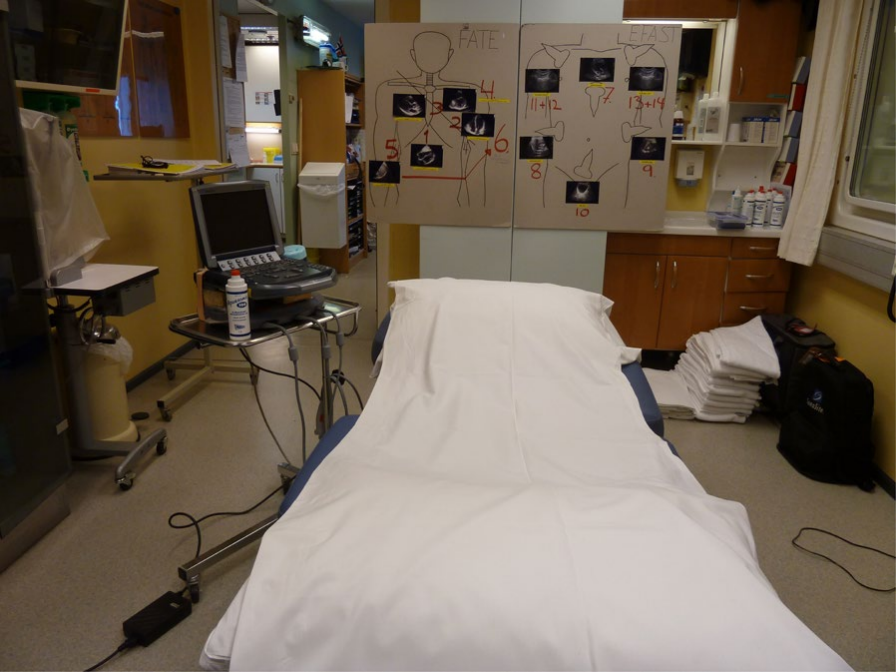
Scanning positions. Offshore hospital bed with M-Turbo ultrasound machine and wall-mounted scanning cards for Focused Assessed Transthoracic Echo (FATE) and extended Focused Assessment with Sonography for Trauma (e-FAST). Ultrasound video clips and images were recorded from all 14 positions. Photo: Nils Petter Oveland

Similarly, the nurse was guided through the examination as described in the FAST protocol and obtained 6-s cineloops for each position. For each volunteer, cineloops were recorded using the curved-array abdominal probe of each of the following positions: (7) subcostal 4-chamber view, (8) peri-hepatic view, (9) peri-splenic view and (10) pelvic view. Finally, cineloops of (11 + 13) lung sliding and (12 + 14) M-mode still images of both lungs were obtained to complete the extended FAST protocol. The setup is summarized in Fig. 3. A research supervisor was present at all times and used a stopwatch to measure the scan time per scanning position. The measurement started when the transducer was placed on the skin and stopped when the onshore expert asked the nurse to store the cineloop or image. When performing the scan, the nurse only received instructions from the onshore expert on how to adjust gain, depth and transducer position. All the cineloops and images where stored on the M-Turbo machine under an alphabetical and numerical code starting with A1, B1, C1, etc. and later transferred to a USB drive.

### Data analysis

The data analysis was binate, with one focus on US image quality and another on scanning time. The image quality of the recorded cineloops (stored on a single USB stick) from both the FATE and e-FAST protocols was blindly evaluated by two independent observers (i.e., an independent US expert panel). A previously described method for evaluating image quality of cineloops [30, 31] was used for the FATE and FAST protocols and a YES/NO answer if lung sliding (cineloop) and seashore sign (M-mode still image) could be identified in the e-FAST lung scans. A five-point scale was used [1 = no visible image (i.e., only air artefacts), 2 = poor image quality with no identifiable anatomical structures, 3 = moderate image quality with partly visible anatomical structures, 4 = good image quality with visible anatomical structures and 5 = excellent image quality with highly visible anatomical structures], with a value of 3 representing the cut-off score for images suitable for interpretation [31]. When calculating the fraction of interpretable images, the 5-point scale was dichotomized, with a score of ≥ 3 indicating that clinically useful information could be extracted from the video clips. The extended part of the FAST protocol was to visualize the pleural line. This step was easily achieved in all volunteers, and scoring the image quality of the lungs was considered less important. Therefore, no image score was assigned from positions 11–14 (lung video clips and M-mode still images). Finally, the total time spent for the FATE and e-FAST protocols was calculated by adding the mean scan time for each position 1–6 and 7–10, respectively.

### Statistical analysis

The image rating scores were analysed using standard descriptive statistics of central tendencies such as median and mean and associated variation as ± standard deviation (SD), interquartile range and minimum–maximum values. The data were outlined graphically as box plots where the length of the box represented the data between the 25 and 75% percentile, the thickened line inside the box showed the median and “whiskers” showed the min and max values. A breakdown of data by acoustic window (i.e., positions 1–6 for FATE and 7–10 for FAST) was done and presented in tables as the mean ± SD. The fraction of interpretable images by acoustic window and protocol (i.e., FATE and FAST) was calculated as the percentage of images with a score of 3 or higher, indicated in tables, figures and text as %^3≥^. Finally, the total scan time was calculated by adding the separate mean scanning times, from probe-on-skin until captured video clip, for positions 1–6, positions 7–10, and positions 11 and 13 for FATE, FAST, and lungs, respectively. All computations were performed using SPSS version 24 (IBM SPSS, Armonk, NY), and data were stored in compliance with current research guidelines.

## Results

**Fig. 4:**
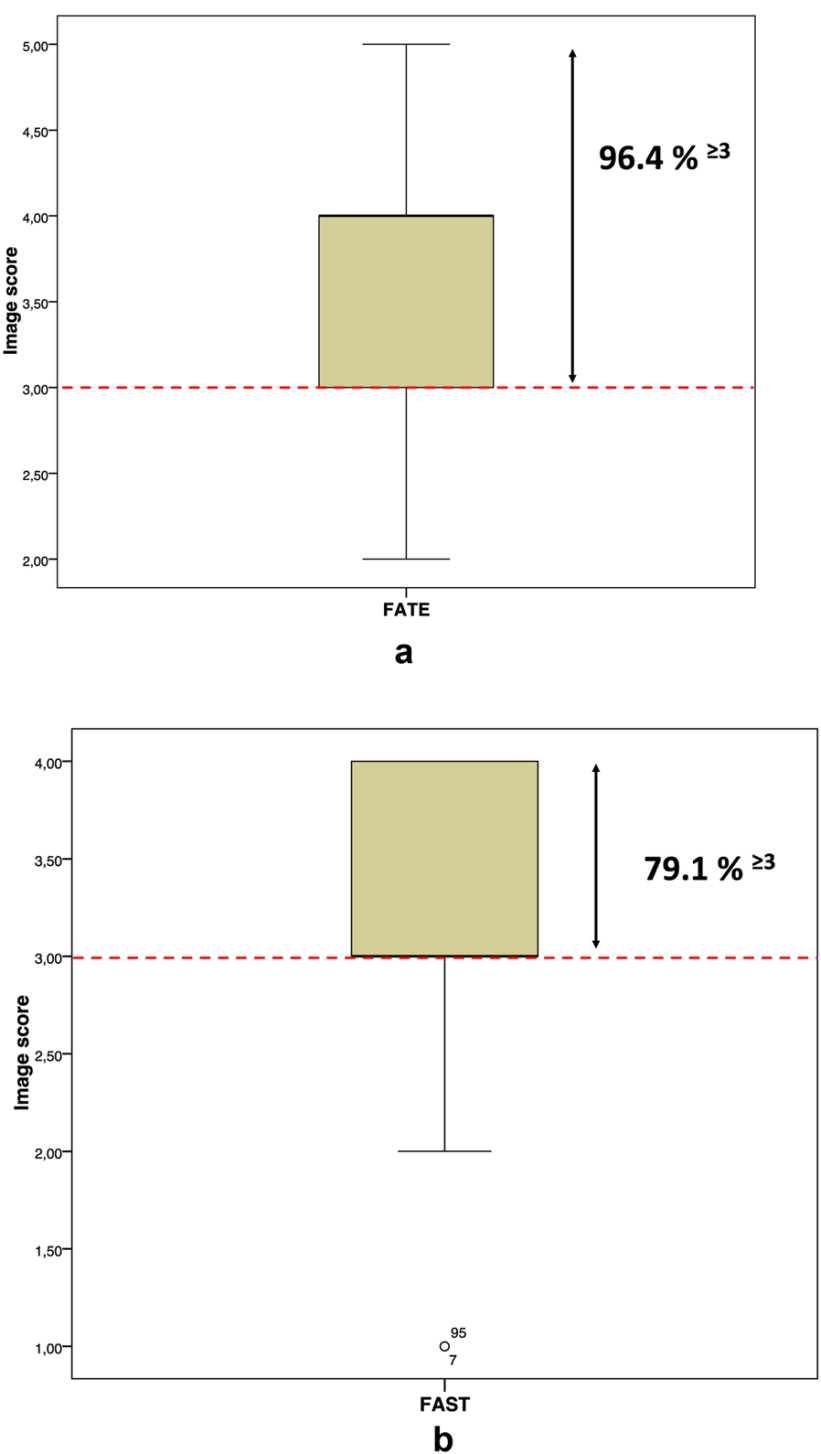
**a** FATE. Box plot showing the median value, interquartile range, and minimum and maximum values. The box plot is dichotomized by the red dotted line with a score of ≥ 3 indicating that clinically useful information could be extracted from the video clips. Of all images from telementored FATE, 96.4% had a quality score of 3 or higher (%^≥3^). **b** FAST. Box plot showing the median value, interquartile range, and minimum and maximum values. The box plot is dichotomized by the red dotted line, with a score of ≥ 3 indicating that clinically useful information could be extracted from the video clips. Of all images from telementored FAST, 79.1% had a quality score of 3 or higher (%^≥3^). Two defined outliers had an image score of 1

All offshore workers that volunteered to be scanned were male with a mean age of 48.7 ± 10.3. The mean weight was 86.1 kg ± 13.4 kg. The image quality score of the cineloops was higher with the FATE protocol than the FAST protocol with medians of 4 and 3, respectively. When comparing the percentage of images suitable for interpretation, 96.4% of the cardiac views (i.e., FATE) using the phased-array transducer and 79.1% of the thoraco-abdominal views (i.e., FAST) using the curved-array transducer had an image score equal to or above 3 (%^≥3)^. No image scores were calculated for the lungs (i.e., e-FAST) as bilateral lung sliding at the pleural line was easily identified in all participants with 100%^≥3^. The median quality scores and fractions of interpretable images for both protocols are outlined as box plots in Fig. 4a, b. Further details about the images’ scores and %^≥3^ by acoustic window when performing telementored FATE and FAST are available in Tables 1 and 2.

**Table 1:** Mean image scores by acoustic window of telementored ultrasound in six different cardio-thoracic windows (FATE)

Acoustic window	Position	*n*	Mean^a^	Standard deviation	%^≥3,b^
Subcostal 4-chamber	1	37	3.49	0.65	92
Apical 4-chamber	2	37	3.54	0.56	100
Parasternal long-axis	3	37	3.32	0.58	97
Parasternal short-axis	4	37	3.32	0.58	95
Pleura right side	5	37	4.19	0.46	100
Pleura left side	6	37	3.70	0.62	95

^a^Image quality scoring system: 1 = no visible image, 5 = excellent image quality

^b^%^≥3^ Percentage of images with a score ≥ 3, which was defined as the cut-off score for images with sufficient quality to extract clinical information, *n* number of scans

**Table 2:** Mean image scores by acoustic window for telementored ultrasound in four different thoraco-abdominal windows (FAST)

Acoustic window	Position	*n*	Mean^a^	Standard deviation	%^≥3,b^
Subcostal 4-chamber	7	37	2.65	0.72	57
Peri-hepatic	8	37	3.35	0.75	84
Peri-splenic	9	37	3.14	0.82	78
Pelvic	10	37	3.73	0.51	97

^a^Image quality scoring system: 1 = no visible image, 5 = excellent image quality

^b^%^≥3^Percentage of images with score of ≥ 3, which was defined as the cut-off score for images with sufficient quality to extract clinical information, *n* number of scans

The mean scan time to complete a full telementored FATE scan comprised six cardio-thoracic views with stored cineloops was 4 min 15 s. Similarly, the mean scan times for FAST comprising four thoraco-abdominal views and bilateral lung scans were 1 min 20 s and 0 min 32 s, respectively. Combined, the mean total scan time to perform a telementored e-FAST was 1 min 52 s. The time measurements for each position were limited to probe-on-skin until image capture. The results for total scan time per protocol, defined as the sum of the mean scan times of each position, are shown in Table 3.

**Table 3:** Total scan time per protocol when performing telementored ultrasound

Examination	Number of scanning positions	*N*	Total scan time
FATE	6	222	4 min 15 s
FAST	4	148	1 min 20 s
Lungs	2^a^	74	0 min 32 s

The protocol including lungs is called extended FAST

*FATE* focus assessed transthoracic echocardiography protocol, *FAST* focused assessment with sonography for trauma

^a^Scanning of each lung to identify lung sliding at the pleural line. The total scan time of the lungs does not include the M-mode scan, *n* number of scans

## Discussion

It is demonstrated in this study that US examinations combined with audio and camera views can be streamed in real-time via a standard internet connection found onboard offshore installations alongside the Norwegian continental shelf. Furthermore, the results show that an expert user can guide an offshore nurse, novel to US, through PoCUS protocols with a high degree of precision and within an acceptable scanning time. Finally, the vast majority of the stored focused heart, lung and abdominal cineloops are of high-quality images suitable for clinical interpretation. These findings are relevant and may enable telementored US to increase diagnostic accuracy with patients in remote locations.

Although it may not be reasonable to consider PoCUS in all clinical settings, a common theme in austere environments is limited access to healthcare resources and imaging capabilities [3, 10]. As one would expect, many of the US applications found useful in-hospital are equally applicable out-of-hospital. Despite the versatile range of diagnostic and procedural applications, implementation of PoCUS in prehospital care has been slow due to both technical and educational barriers. It is thus important that any equipment carried to a remote location is lightweight, durable and rugged [32]. Many of the current machines are portable and battery-powered and can be brought to the patient regardless of location [31]. They are increasingly cheap, robust and produce high-quality images. Technological advances have led to truly hand-carried PoCUS devices [33]. Another important technical feature of these machines is the capacity for the connections to different platforms, systems and applications [34, 35]. A systematic review by Gopaul et al. [36] lists the lack and cost of formal US training as the main barriers to implementing US in resource-limited settings. Current World Health Organization (WHO) recommendations for US training are quite extensive and involve 300–500 US scans per physician to achieve an acceptable skill level [37]. Our study shows that this educational barrier can be lowered by having experts connect to novice users and guide them through PoCUS protocols. By simply knowing how to operate the on/off button, gain and depth adjustments and save function on the M-Turbo machine, the offshore nurse procured cineloops and images of diagnostic value (score ≥ 3) in 96.4% of the cardiac views and 79.1% of the thoraco-abdominal views. These results clearly indicate that telementored US has the potential to augment clinical decision-making when used as an adjunct to the standard physical examination and perhaps being the only realistic imaging modality available to offshore patients.

In trauma care, it is important to detect the presence of major thoracic and abdominal fluid collection in patients with haemorrhagic shock. The FATE [38] and FAST [39] protocols were developed to efficiently diagnose or rule out a variety of life-threatening conditions. FAST is the basic building block for PoCUS and one of the most studied US tools for haemodynamically unstable patients. Not only it is an effective scan to diagnose free fluid in the thoraco-abdominal cavities and pericardial sac, but it can also be performed quickly and at bedside [39]. The results show that telementored FAST took only 1 min and 20 s, with an additional 32 s to scan the lungs (i.e., extended version of FAST). Although more comprehensive (4 min and 15 s), FATE is one of the most valuable scans for cardiac function, potentially revealing the causes of cardiac arrest and circulatory shock. This protocol has been developed to define the aetiology of circulatory collapse and more specifically to identify reversible causes during ongoing resuscitation (i.e., diagnosing fluid in the pericardial sac or pleural space and evaluating the size, shape and function of the ventricles) [25, 28]. For critically ill and injured patients, time is always critical, and many deaths are preventable if reversible causes are recognized and treated expeditiously [40, 41]. In remote settings, it is important that patients are triaged and transported to the correct level of care. PoCUS examinations performed on-site within a few minutes are thus clinically extremely attractive [3]. The mean scan time for the FATE protocol was longer than FAST but involved obtaining six views rather than four (Table 3). The time recorded in our study was from skin contact to cineloop or image capture, not the time in between the different scanning positions. The reason for this was that the volunteers in this study were on duty when being scanned and consequently had to be available for phone calls and urgent matters if needed. As a result, several of the scanning sessions were disjoined and for this reason the total scan time was not recorded. Total scan time would, therefore, be slightly longer, but both protocols can be performed in a time frame fully comparable to many other diagnostic procedures (e.g., CT, ECG, heart and lung auscultation, arterial and venous blood sampling).

The diagnostic quality of the cineloops obtained from the FATE protocol exceeded those from the FAST protocol, which was a surprise to us, as cardiac scans have been perceived as more cumbersome and difficult to acquire. When looking at the image score by acoustic window for FAST (Table 2), the subcostal 4-chamber view stands out with a much lower %^≥3^ score (57%) than the other three views (84%, 78% and 97%, respectively), bringing the total %^≥3^ score (79%) for this protocol down considerably. The subcostal 4-chamber view is also part of the FATE protocol (Table 1), but the ≥ 3 score was 92%. This discrepancy could be explained by the different transducers used for FAST and FATE. The transducer for the FATE protocol uses phased-array ultrasonics where the US beam can be swept electronically without moving the probe (i.e., beam steering technology). This difference makes it better suited for visualizing a beating heart compared to the more fixed US beam emitted from the curved-array transducer used for the FAST protocol. As an alternative, the phased-array transducer can also be used for FAST. The total FAST %^≥3^ score in our data (Fig. 4b) could, therefore, theoretically increase from 79.1 to 88% if the probes were switched for the subcostal 4-chamber view. However, looking at all data combined, diagnostic information can be extracted from 87.8% of all images (%^≥3)^. Furthermore, our study shows that evaluating the lungs is rapid and feasible in all patients. This finding is important as evaluating the pleural line for horizontal sliding (i.e., lung sliding) could distinguish between a normal lung with lung sliding and a pneumothorax without lung sliding [29, 42]. This diagnostic amendment has been added to the standard version of FAST, hence the name e-FAST [43]. In our study, scores were not given for the lung scans (video for lung sliding and M-mode image of the pleural line), but these images and videos were evaluated for whether presence of lung sliding and seashore sign (M-mode) were visible. The expert user found that the pleural line and lung sliding were visible in all images and videos obtained (100%). This high feasibility is likely because the pleural line is located superficially (i.e., close to the anterior chest) in most people, regardless of body state, thus making it easy to visualize with US. However, there are some limitations to lung US, such as the presence of dressings or subcutaneous empyema [44].

Previous literature on telementored US discuss feasibility, technicality and outcomes of having a remote expert guiding novice users in different PoCUS protocols. A selection of relevant studies is shown in Table 4. The research includes a variety of locations where US images had been transferred. The majority of the publications are feasibility studies on healthy volunteers, whereas studies looking at real patients seem to, for practical reasons, take place at hospitals [17, 22, 45]. A study by McBeth et al. [46] also used a phantom to assess the participants’ ability to identify simulated pathology. FAST and e-FAST are the most commonly studied protocols, but in a few publications, the US examinations were adapted to meet specific patient needs [22, 47]. The communication equipment used is described as basic and can be bought off-the-shelf, such as laptops with a web camera, mobile phones with FaceTime™ or similar software apps and head-mounted cameras (e.g., Go-Pro™) [17, 22, 46–49]. One exception, similar to our study, is a publication by Olivieri et al. [45] where an already available advanced telecommunication system was used. In all studies, a recurrent theme is that data transmission (i.e., real-time transfer of US and video) is limited by network availability and internet access [3]. Therefore, telementored US may not be suitable for all geographic areas. A systematic review by Marsh-Failey et al. [50] suggests a minimum bandwidth for image transfer of 500 kbps, but the studies described in Table 4 use either a 3G or 4G data network or public wireless network (WiFi), with no significant delay in image transfer reported. In our study, we used a fibre-optic internet line that was part of a deep-sea cable stretched out on the seabed from Statfjord C to an onshore relay station. We did not experience any problems with the transfer of US images from the M-Turbo machine or the roof camera from the hospital bay with both split-screens displayed on the onshore computer (Fig. 2). In contrast, the onshore US expert perceived all PoCUS examinations in real-time with no delay in audio or video signals. The nurse performing the scans could, as described, at all time see the US expert in real-time on the Medical unit screen. The possible impact this might have on the scan performance has not been investigated or analysed. As the videoconference setup allowed for bidirectional video, disabling this function was not considered. It was not crucial for the nurse being able to see the US expert as all instructions given were verbally. The US expert has however stated that he considered it a positive element in guiding process, that both pars could see each other real-time.

**Table 4:** Selection of articles showing the use and effect of tele-ultrasound

References	Study design	Type of equipment	No of scans performed	Protocols/anatomy scanned	Main findings
Dyer et al. [49]	Pilot study	Sonix OP Bidirectional videoconference system	20	FAST e-FAST	Installing a tele-ultrasound system for major trauma cases was found to be technically and clinically feasible. The remote expert was able to diagnose pathology with FAST and e-FAST. The technology was also found to enhance ultrasound education and occasionally facilitated important clinical decision-making
Mc Beth et al. [48]	Case series	Sonosite 180 Head camera Laptop w/Skype	N/A	e-FAST	With the use of basic, low-cost cellular networks, it is possible to conduct telementored trauma sonography and produce images of excellent diagnostic quality
Biegler et al. [17]	Feasibility study	Sonosite NanoMaxx Head camera Computer w/Skype	26	Lung	Ultrasound together with simple informatics technologies permits remote telementored ultrasonography as long as internet is available. An ultrasound expert could guide a novice ultrasound user in performing lung ultrasound for detection of pneumothorax post-chest tube removal
Mc Beth et al. [46]	Feasibility study	Sonosite NanoMaxx Head camera Laptop	90	FAST e-FAST	A remote telementored ultrasound system was easy to implement, and with wireless internet, allowed a remote expert to instruct a novice user to obtain diagnostic images for interpretation
Kolbe et al. [22]	Pilot study	Sonosite Titan Laptop w/Skype	132	Foetus/pregnancy Abdomen	Local practitioners in rural areas can, after didactic training, perform POCUS under teleguidance. The implementation of POCUS in a rural village led to a change in management of about half of the patients scanned
Robertson et al. [47]	Feasibility study	Sonosite M-turbo Apple iPhone Apple MacBook Pro	63	Internal jugular vein, lung, heart, bladder	Low-cost commercially available equipment can be used for real-time mentored acquisition and interpretation of high-quality US images that are clinically useful
Olivieri et al. [45]	Pilot study	Sonosite X-porte Tele-ICU program w/camera	40	Heart Lung	After a 60-min training session, a remotely located tele-intensivist could guide a novice US user in performing heart and lung ultrasound. Remote telementored ultrasound could be used to evaluate patients in respiratory failure and/or shock in situations where US-proficient providers are not available at the bedside

**Fig. 5:**
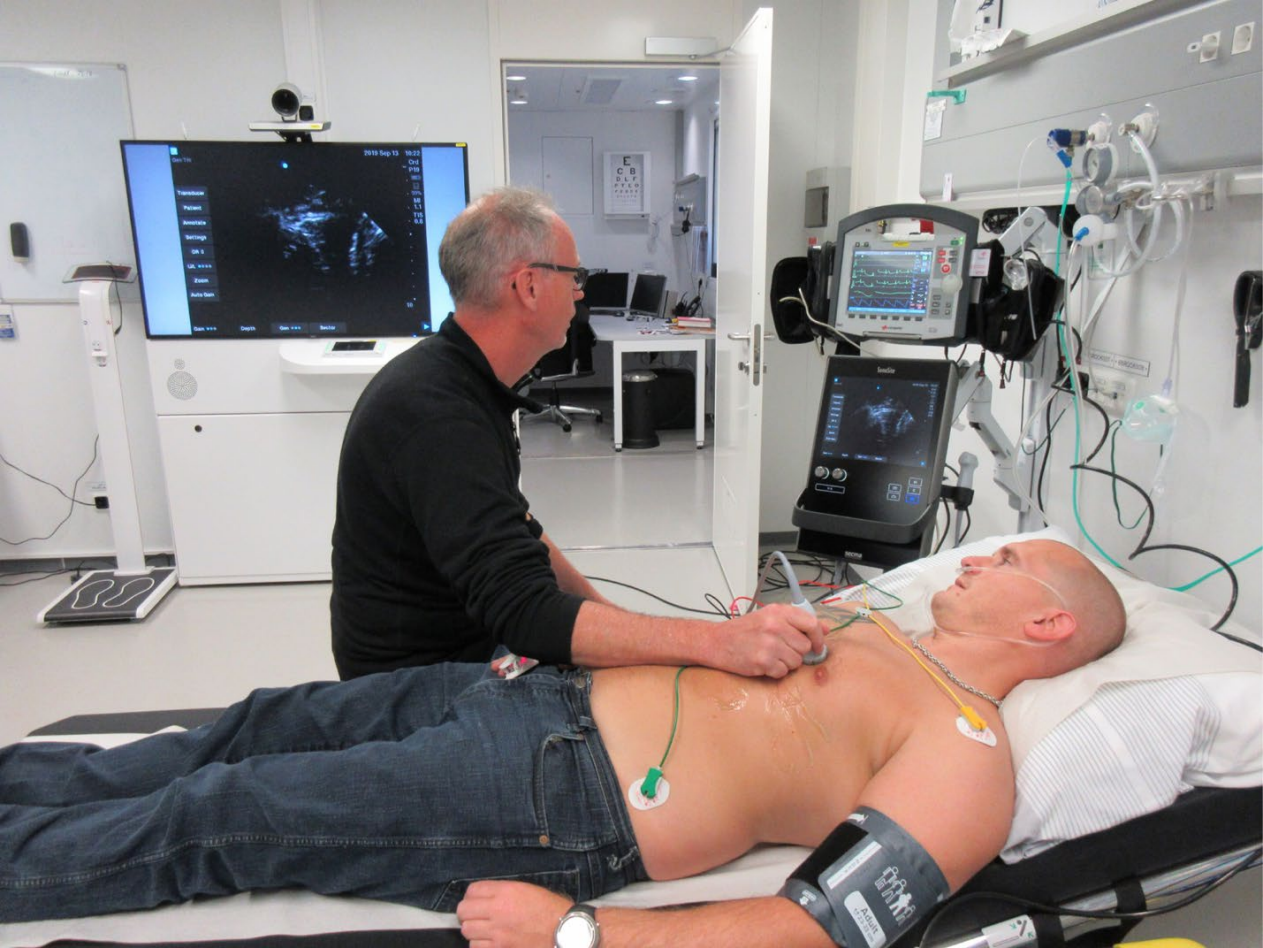
Telementored ultrasound from the Aasta Hansteen platform in the Norwegian Sea. This oil installation is 300 km from the Norwegian coast, remote from other installations and in an area with extreme weather conditions. Its modern hospital is equipped with a wall-mounted SII Sonosite™ ultrasound machine (bottom) and Corpuls 3™ monitor/defibrillator machine (top), connected to the Medical unit in the background. Telementored ultrasound and live transfer of patient data such as ECG, blood pressure and saturation, make it possible for physicians to monitor and diagnose critically ill and injured patients from onshore hospitals in Norway. Photo: Svein Stalheim

Remote oil and gas operations present a multitude of health risks. However, they can be kept as low as reasonably practicable by implementing a number of strategies, such as health risk assessments, medical emergency response planning including medevac, healthcare practitioner competency requirements, remote medical support and telemedicine [5, 6, 12]. To our knowledge, we are the first to explore the use of telementored US in an offshore setting. A few oil companies have, since the data collection period in 2012, installed US in their hospitals, but these installations have almost exclusively been in extremely remote locations (i.e., sites where medical evacuation to a hospital can never be achieved within 4 h, even in the best of circumstances). Increasingly, the energy industry is operating in these environments with the goal of protecting the health of the offshore workers at an equal standard as their non-remote counterparts. As part of their remote healthcare strategy, Equinor ordered an investigation of their offshore medical healthcare systems to describe the use of their SAR helicopters and register the patients’ diagnoses or symptoms leading to emergency medevac. This process resulted in a prospective study by Østerås et al. [13] looking at baseline characteristics of all patients (*n* = 382) evacuated by SAR from three Equinor offshore installations in the North Sea over a time period of 2 years. The top three diagnoses or symptoms were chest pain (*n* = 102), abdominal pain (*n* = 75) and trauma (*n* = 68), making up 65% of all patients. PoCUS may be used as a diagnostic tool for all these conditions and some others on the list [e.g., cardiac arrhythmia (*n* = 10), breathing difficulties (*n* = 7), cardiac arrest (*n* = 4) and obstetrics (*n* = 1)]. Altogether, telementored US could possibly be useful in up to 71% of patients being evacuated from an offshore platform. Furthermore, out of all SAR missions, 71% were a code yellow. This code is used in situations where a patient’s condition is not immediately life-threatening (i.e., code red) but often undetermined and may deteriorate. These patients are often evacuated if the offshore nurse and onshore physician feel that further examination and early treatment are necessary. Some patients are even evacuated as a preventive action or simply because of limited access to diagnostic capabilities at the offshore hospitals. There are high costs involved in using the SAR service for medical evacuations, and by adding PoCUS, the nurses might be able to identify and monitor numerous conditions that safely can be treated on-site. Hence, oil and gas companies may prevent health complications, minimize unnecessary medical evacuations (code yellow), facilitate necessary ones (codes yellow and red) and optimize care during transfer (codes yellow and red) if they implement telementored US offshore. Figure 5 shows the setup of an US machine connected to the Medical unit at an offshore hospital today.

From a more global perspective, the World Health Organization (WHO) states that diagnostic imaging is a necessary procedure for accurately treating at least 25% of the worlds’ population and that X-ray and US alone can meet over 90% of imaging needs [51]. Therefore, they recommend X-ray and US be available for all patients in primary healthcare settings [52]. However, US technology has several advantages compared to X-ray. It is cheaper, portable, battery-powered, dynamic and does not involve ionizing radiation [16]. For all these reasons, telementored US should be part of a global commitment to provide clinical support and overcome geographical barriers with the overall aim of improving health outcomes.

## Limitations

First, this study was a small quality assessment study with a limited number of healthy participants (*n* = 37) where all volunteers were male. Second, the telementored US examinations were between the same offshore nurse and onshore expert in all cases which most likely improved the scan time and image quality over the course of the study. However, the data collection was done over a short period of time and for that reason we chose not to analyse the progression potentially reflected in the improved scan time and image quality. Third, the transmission times for audio and video signals (both US and roof camera) were not calculated, but only perceived by the onshore expert to be in real-time without any delays in synchronous streaming. Finally, we used an advanced medical communication unit (Medical unit) connected to the internet via a high-speed fibre-optic cable and did not encounter any issues with the technical equipment during the 2 days of scanning. For these four reasons, the generalizability of our results may be diminished. However, our data analysis is based on a total of 518 telementored US scans, all performed in exactly the same way with the same collaborating nurse and physician. Previous studies have not described the need to use advanced communication systems but rather various cheap and commercially available communication hardware and software (Table 4). Furthermore, a systematic review shows that even a bandwidth speed as low was 500 kbps could be sufficient for synchronous streaming with good image quality [50]. In our study, the US images and videos were retrieved from the US machine to a USB stick and reviewed retrospectively; hence, image degradation due to bandwidth speed was not an issue when evaluating image quality scores. Last, we want to comment on the fact that the data collection was carried out several years ago. There has not been much change with regard to the use of offshore telementored ultrasound and the equipment used in this study is still in use today. We therefore believe that the data are still valid and applicable.

## Conclusion

Telementored US using existing communication and network infrastructure available at offshore oil and gas installations in the North Sea is feasible and allows real-time sharing of US cineloops and images. Remotely located experts onshore can guide inexperienced offshore nurses through different PoCUS examinations with ease, such as FATE and e-FAST. Finally, the vast majority of telementored US images and cineloops procured by novices are of high enough quality to visualize relevant anatomical landmarks and to extract diagnostic information. Future research should focus on clinical outcomes of implementing telementored US in remote locations.

## Data Availability

All the ultrasound cineloops and images were stored on the M-Turbo machine under an alphabetical and numerical code starting with A1, B1, C1, etc. and later transferred to a USB drive. The time per scanning position was written down on paper using the same alphabetical and numerical coding system. All the data were handled and analysed anonymously. All storage of written and electronic data was done according to good clinical practices and the authors’ university guidelines.

## Abbreviations

US: Ultrasound
CT: Computerized tomography
MRI: Magnetic resonance imaging
PoCUS: Point-of-care ultrasound
RTMUS: Remote telementored ultrasound
FAST: Focused assessment with sonography for trauma
e-FAST: Extended focused assessment with sonography for trauma
FATE: Focused assessed transthoracic echocardiography
SAR: Search and rescue
Medevac: Medical evacuation
ECG: Electrocardiography
CRP: C-reactive protein
WHO: World Health Organization
NSD: Norwegian Centre for Research Data
REK: Research Ethics Committees
VGA: Video graphics array
